# GM-CSF and IL-3 Modulate Human Monocyte TNF-α Production and Renewal in *In Vitro* Models of Trained Immunity

**DOI:** 10.3389/fimmu.2016.00680

**Published:** 2017-01-16

**Authors:** Francesco Borriello, Raffaella Iannone, Sarah Di Somma, Stefania Loffredo, Eloise Scamardella, Maria Rosaria Galdiero, Gilda Varricchi, Francescopaolo Granata, Giuseppe Portella, Gianni Marone

**Affiliations:** ^1^Department of Translational Medical Sciences, University of Naples Federico II, Naples, Italy; ^2^Center for Basic and Clinical Immunology Research (CISI), University of Naples Federico II, Naples, Italy; ^3^Institute of Experimental Endocrinology and Oncology “Gaetano Salvatore” (IEOS), National Research Council (CNR), Naples, Italy

**Keywords:** innate immune memory, immunometabolism, inflammation, monocyte, macrophage, TLR, GM-CSF, IL-3

## Abstract

GM-CSF and IL-3 are hematopoietic cytokines that also modulate the effector functions of several immune cell subsets. In particular, GM-CSF and IL-3 exert a significant control on monocyte and macrophage effector functions, as assessed in experimental models of inflammatory and autoimmune diseases and also in human studies. Here, we sought to investigate the mechanisms and the extent to which GM-CSF and IL-3 modulate the pro-inflammatory, LPS-mediated, activation of human CD14^+^ monocytes taking into account the new concept of trained immunity (i.e., the priming stimulus modulates the response to subsequent stimuli mainly by inducing chromatin remodeling and increased transcription at relevant genetic loci). We demonstrate that GM-CSF and IL-3 priming enhances TNF-α production upon subsequent LPS stimulation (short-term model of trained immunity) in a p38- and SIRT2-dependent manner without increasing *TNF* primary transcript levels (a more direct measure of transcription), thus supporting a posttranscriptional regulation of TNF-α in primed monocytes. GM-CSF and IL-3 priming followed by 6 days of resting also results in increased TNF-α production upon LPS stimulation (long-term model of trained immunity). In this case, however, GM-CSF and IL-3 priming induces a c-Myc-dependent monocyte renewal and increase in cell number that is in turn responsible for heightened TNF-α production. Overall, our results provide insights to understand the biology of monocytes in health and disease conditions in which the hematopoietic cytokines GM-CSF and IL-3 play a role and also extend our knowledge of the cellular and molecular mechanisms of trained immunity.

## Introduction

GM-CSF and IL-3 are hematopoietic cytokines that bind to heterodimeric receptors composed of a β common subunit (CD131) and specific α subunits (CD116 and CD123, respectively) (1, 2). These cytokines are produced by many immune and non-immune cells and are important mediators of emergency myelopoiesis, albeit their role in steady-state myelopoiesis is dispensable (3–6). GM-CSF and IL-3 also modulate the effector functions of several mature immune cell subsets and have been shown to play a role in inflammatory and autoimmune diseases (7–11). For example, GM-CSF promotes the maturation and effector functions of myeloid cells (e.g., monocytes and neutrophils) and plays a protective role in an experimental model of acute sepsis (12). On the other hand, IL-3 exacerbates myelopoiesis and incites a cytokine storm (i.e., TNF-α, IL-1β, and IL-6) that is detrimental to the host in the same sepsis model (6). In an animal model of multiple sclerosis (experimental autoimmune encephalomyelitis), conditional deletion of *Csf2rb* (the mouse ortholog of *CD131*) in CCR2^+^ monocytes abrogates a pathogenetic signature that is required for disease development (7, 8). Interestingly, GM-CSF modulates several functions of human CD14^+^ monocytes (13–15), including cytokine production upon LPS stimulation. We have recently shown that GM-CSF and IL-3 synergize with IL-4 to enhance the production of the IL-4-responsive chemokine CCL17/TARC by human CD14^+^ monocytes (16). Thus, GM-CSF and IL-3 exert a significant control on monocyte effector functions. The mechanisms and the extent to which GM-CSF and IL-3 modulate the pro-inflammatory (e.g., LPS-mediated) activation of human CD14^+^ monocytes are still unclear.

Most of the functional and molecular studies on monocyte and macrophage effector functions have relied on the activation of these cells by discrete stimuli. However, it is now clear that monocytes and macrophages are exposed to a plethora of stimuli *in vivo* that eventually determines their functional specialization (17). Moreover, these cells may be exposed to different and sometimes opposing stimuli in a sequential manner, and the priming stimulus may exert a significant control over the response to subsequent stimuli. This phenomenon, which is referred to as trained immunity or innate immune memory, has been reported in several organisms and with a variety of stimuli, including toll-like receptor (TLR) agonists and cytokines (18, 19). The *in vitro* and *in vivo* experimental approaches employed so far consist of either priming followed shortly after (e.g., the next day) by activation with the same or a different stimulus (short-term model) (20–25) or priming followed by prolonged resting (e.g., 5–6 days or several weeks) before stimulation (long-term model) (26–30). Most of these models point to chromatin remodeling as a mechanism to retain memory of the priming stimulus. Critically, *in vivo* evidence of trained immunity has also been obtained in humans (27). Nevertheless, we still lack complete knowledge of the mediators and the cellular and molecular mechanisms of trained immunity. The answer to these questions is poised to have broad implications for understanding the pathogenesis of and defining new therapeutic strategies for infectious and chronic inflammatory diseases as well as vaccine design (19, 31, 32).

Here, we sought to investigate whether GM-CSF and IL-3 modulate the pro-inflammatory, LPS-mediated, activation of human CD14^+^ monocytes taking into account the new concept of trained immunity. We found that in a short-term model of trained immunity, GM-CSF and IL-3 priming enhances LPS-induced TNF-α production in a SIRT2- and p38-dependent manner by increasing TNF-α protein levels without significantly modifying RNA levels. At variance with this, in a long-term model of trained immunity, GM-CSF and IL-3 priming induces monocyte renewal in a c-Myc-dependent manner, thereby enhancing LPS-induced TNF-α production. Our results uncover new mediators (GM-CSF and IL-3) in *in vitro* models of trained immunity that modulate TNF-α production through transcription-independent mechanisms.

## Materials and Methods

### Cell Isolation and Culture

The study protocol involving the use of human blood cells was approved by the Ethics Committee of the University of Naples Federico II. Cells were isolated from buffy coats of healthy donors. Blood was layered onto Histopaque-1077 (Sigma-Aldrich) and mononuclear cells were collected at the interface. Monocytes were further purified with anti-CD14 Microbeads (Miltenyi Biotec). Purity of cell preparations was >95% as assessed by flow cytometry. Cells were cultured in cIMDM-5 [IMDM, 5% FCS, 1× non-essential amino acids, 1× UltraGlutamine, 25 mM HEPES, 5 µg/mL gentamicin (Lonza)] in 96-well flat-bottom plates (10^5^ monocytes/well) in a final volume of 250 µL. For experiments involving flow cytometry, cells were cultured in suspension (1.5 mL tubes) in cIMDM-5 at a concentration not greater than 2 × 10^6^ cells/mL, then spun down and collected for subsequent experiments.

Cells were treated with different combinations of: LPS (*Escherichia coli* 026:B6) 10 ng/mL (Sigma-Aldrich), IL-3 5 ng/mL (Peprotech), M-CSF 25 ng/mL, GM-CSF 5 ng/mL (Miltenyi Biotec), P3CSK4 10 ng/mL, Poly(I:C) 1 µg/mL, flagellin 10 ng/mL, imiquimod 1 µg/mL, ODN2006 1 µM (Invivogen), BAY11-7082 1 µM, SP600125 2 µM, rapamycin 50 and 250 nM, torin1 10 and 50 nM, AGK2 10 µM, APO866 0.1, 1, and 10 nM, TG101348 125, 250, and 500 nM (Selleckchem), U0126 2 µM, LY294002 10 µM, SB203580 2 µM (Cell Signaling Technology), 10058-F4 40 µM, EX-527 500 nM (Tocris Bioscience), 2-Deoxy-d-Glucose 1 mM, Etomoxir 40 µM, BAY 85-3934 1 µM, CAY-10585 10 µM (Cayman Chemical), nicotinic acid 10 µM (Sigma-Aldrich), Pyridone 6 100 nM (BioVision), Trichostatin A (TSA) 5 nM (Calbiochem).

### ELISA and Nicotinamide Adenine Dinucleotide (NAD) Quantification

Cytokine concentrations in cell-free supernatants or total protein lysates (0.1% Triton X-100) were measured using ELISA kits for TNF-α (R&D Systems), IL-1β, and IL-6 (eBioscience). Standard curves were generated with a Four Parametric Logistic curve fit and data were analyzed using MyAssays Analysis Software Solutions (www.myassays.com). Cytokine levels in protein lysates were normalized on total protein concentrations as determined by Bradford protein assay (Biorad) and expressed as picogram of cytokine per microgram of total protein. Intracellular NAD levels were measured using the colorimetric assay NAD/NADH Quantitation assay (Sigma-Aldrich) according to the manufacturer’s protocol and expressed as fold over control.

### Phospho-Flow Cytometry

Monocytes were collected, rested in cIMDM-0.5 (0.5% FCS) for 1 h, and stimulated with LPS for the indicated times. Then, cells were fixed with 1.5% paraformaldehyde (EM-grade, Electron Microscopy Sciences) and permeabilized with absolute ice-cold methanol. Cells were stained (60 min at room temperature) in PBS + 10% human AB serum (Lonza) + 0.05% NaN_3_ with the following antibodies: anti-human-phospho-p38 PE (T180/Y182) (3D7, dilution 1:50), anti-human-phospho-p44/42 (ERK1/2) PE (T202/Y204) (19762, dilution 1:50) (Cell Signaling Technology). Samples were acquired on MACSQuant Analyzer 10 (Miltenyi Biotec) and analyzed using FlowJo v10. Doublets and debris were excluded from the analysis. Data are expressed as percentage of positive cells and median fluorescence intensity (MFI).

### Fluorescence, Time-Lapse, and High-Content Microscopy

Microscopy experiments were performed with the Operetta High-Content Imaging System (PerkinElmer). Monocytes were cultured in 96-well black CellCarrier plates (PerkinElmer). For fluorescence microscopy, cells were fixed with 1% paraformaldehyde (EM-grade, Electron Microscopy Sciences) and permeabilized with PBS + 0.1% Triton X-100. Then, actin was stained with rhodamin phalloidin and nuclei with Hoechst 33342 (Thermo Fisher Scientific) according to manufacturer’s protocol. Representative images were taken with a 60× objective. For time-lapse microscopy, monocytes were cultured for 6 days at controlled temperature (37°C) and CO_2_ (5%). Over this time window, digital phase contrast images of 15 fields/well were taken every 60 min with a 20× objective. Representative videos of single fields are shown for each experimental condition. To quantify cell confluency, brightfield images of 15 fields/well were taken on days 1, 4, 7, and 8 with a 20× objective. PhenoLOGIC (PerkinElmer) was employed for image segmentation and to calculate the cell-free area (*b*) and the area covered by cells (*a*). Cell confluency was calculated as a proportion of cell area over total cell area using the following formula: [*a*/(*a* + *b*)] × 100.

### Phagocytosis Assay

Monocytes were cultured in 96-well black CellCarrier plates (PerkinElmer) and stimulated for 18 h without or with M-CSF, IL-3, and GM-CSF. Then, pHrodo Green *E. coli* BioParticles (Thermo Fisher Scientific) were added for 1 h (150 µg/mL final concentration) and actin and nuclei were stained as indicated above. Fluorescence microscopy images of 15 fields/well were obtained with the Operetta High-Content Imaging System (PerkinElmer). Cells were identified based on nuclei and actin staining. Then, the percentage of cells (*a*) that had phagocytosed *E. coli* BioParticles (i.e., cells emitting in the FITC channel) over total cells (*b*) was calculated using the following formula: (*a*/*b*) × 100.

### RNA Isolation and Real Time RT-PCR

RNA was extracted using TRIzol (Thermo Fisher Scientific) and reverse-transcribed (500 ng) using SuperScript III Reverse Transcriptase (Thermo Fisher Scientific). Real-time RT-PCR was performed by using Universal SYBR Green Supermix (Bio-Rad) on CFX96 Real-time detection system (Bio-Rad). Relative quantification of gene expression was calculated by the Δ*Ct* (relative expression × 10^4^) method. Each *Ct* value was normalized to the respective *ubiquitin C* (*UBC*) *Ct* value. The following primer pairs were used: *TNF* mRNA forward 5′-tctccttcctgatcgtggca-3′; *TNF* mRNA reverse 5′-cagcttgagggtttgctacaac-3′; *TNF* primary transcript forward 5′-taagggtgactccctcgatgt-3′; *TNF* primary transcript reverse 5′-ccaaacccaaacccagaatta-3′; *SIRT2* forward 5′-cccaaccccagcaaatctctaa-3′; *SIRT2* reverse 5′-ggctggtagagatgcctgtt-3′; *MYC* forward 5′-attctctgctctcctcgacg-3′; *MYC* reverse 5′-agcctgcctcttttccaca-3′; *MAFB* forward 5′-gtatgaaccgcatggtgcttg-3′; *MAFB* reverse 5′-ctgttgcggcaggtttgatt-3′; *UBC* forward 5′-ggtcgcagttcttgtttg-3′; *UBC* reverse 5′-gatggtcttaccagtcaga-3′.

### Statistical Analysis

Statistical analysis was performed with Prism 6 (GraphPad Software). *P* values were calculated as indicated in figure legends. *P* < 0.05 was considered significant.

## Results

### GM-CSF and IL-3 Priming Enhances LPS-Induced TNF-α Production

**Figure 1:**
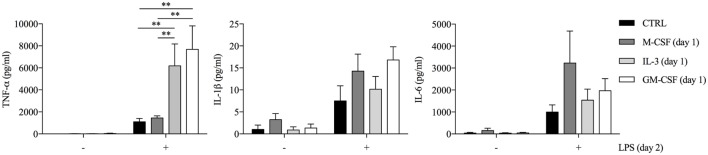
**GM-CSF and IL-3 priming enhances TNF-α production upon LPS stimulation in a short-term model of trained immunity**. Human CD14^+^ monocytes were left unprimed (CTRL) or primed with M-CSF, IL-3, and GM-CSF for 18 h (day 1), then washed, and left untreated or stimulated with LPS for 16–18 h (day 2). TNF-α, IL-1β, and IL-6 levels were assessed by ELISA in cell-free supernatants. Data are shown as mean + SEM of seven independent experiments. **P < 0.01 determined by repeated measure two-way ANOVA with Sidak’s *post hoc* test.

In order to evaluate the effect of GM-CSF and IL-3 on the pro-inflammatory LPS-mediated activation of human CD14^+^ monocytes, we simultaneously treated these cells with LPS and GM-CSF or IL-3 and then assessed the production of TNF-α, IL-1β, and IL-6. To our surprise, neither GM-CSF (Figure S1A in Supplementary Material) nor IL-3 (Figure S1B in Supplementary Material) modulated cytokine production in response to LPS. Since several stimuli may act as priming stimuli (18, 19), we reasoned that GM-CSF and IL-3 could prime monocytes for increased responsiveness to LPS. Monocytes were left untreated (CTRL) or treated for 18 h with M-CSF, IL-3, or GM-CSF, washed and then stimulated with LPS for additional 16–18 h (short-term model of trained immunity). Interestingly, priming with GM-CSF or IL-3 increased LPS-induced TNF-α production, while IL-1β and IL-6 were marginally affected (Figure 1) and no IL-12p70 production was detected (data not shown). Furthermore, GM-CSF and IL-3 priming did not enhance *E. coli* phagocytosis (Figure S2 in Supplementary Material). To verify whether the priming effect was shared among different TLRs, we stimulated primed monocytes with LPS (TLR4 agonist), P3CSK4 (TLR2 agonist), Poly(I:C) (TLR3 agonist), flagellin (TLR5 agonist), imiquimod (TLR7 agonist), or ODN 2006 (TLR9 agonist). The priming effect was more pronounced when monocytes were stimulated with LPS, although a trend toward an increase in TNF-α production was also observed with P3CSK4 (Figure S3 in Supplementary Material). Finally, a time-course analysis revealed that at least 3 h (for IL-3) or 6 h (for GM-CSF) of priming were required to observe increased TNF-α secretion upon LPS stimulation (Figure S4 in Supplementary Material).

To gain insights into the molecular mechanisms of monocyte priming, we selectively treated monocytes during the priming phase with inhibitors of pathways that are known to modulate monocyte and macrophage activation: JAK2 (TG101348), NF-κB (BAY 11-7082), c-Myc (10058 F4), HIF-1α (CAY10585), PHD (BAY 85-3934), PI3K (LY294002), MEK1/2 (U0126), p38 (SB203580), JNK (SP600125), hexokinase (2-Deoxy-d-Glucose, inhibitor of glycolysis), CPT1 (etomoxir, inhibitor of fatty acid β-oxidation). The readout of these experiments was the modulation of TNF-α production by primed monocytes without affecting unprimed cells. Most of the inhibitors had no or negligible effects on primed cells, while TG101348 reduced TNF-α production by both primed and unprimed cells (Figure S5 in Supplementary Material).

### GM-CSF and IL-3 Priming Modulates TNF-α Production at the Posttranscriptional Level

**Figure 2:**
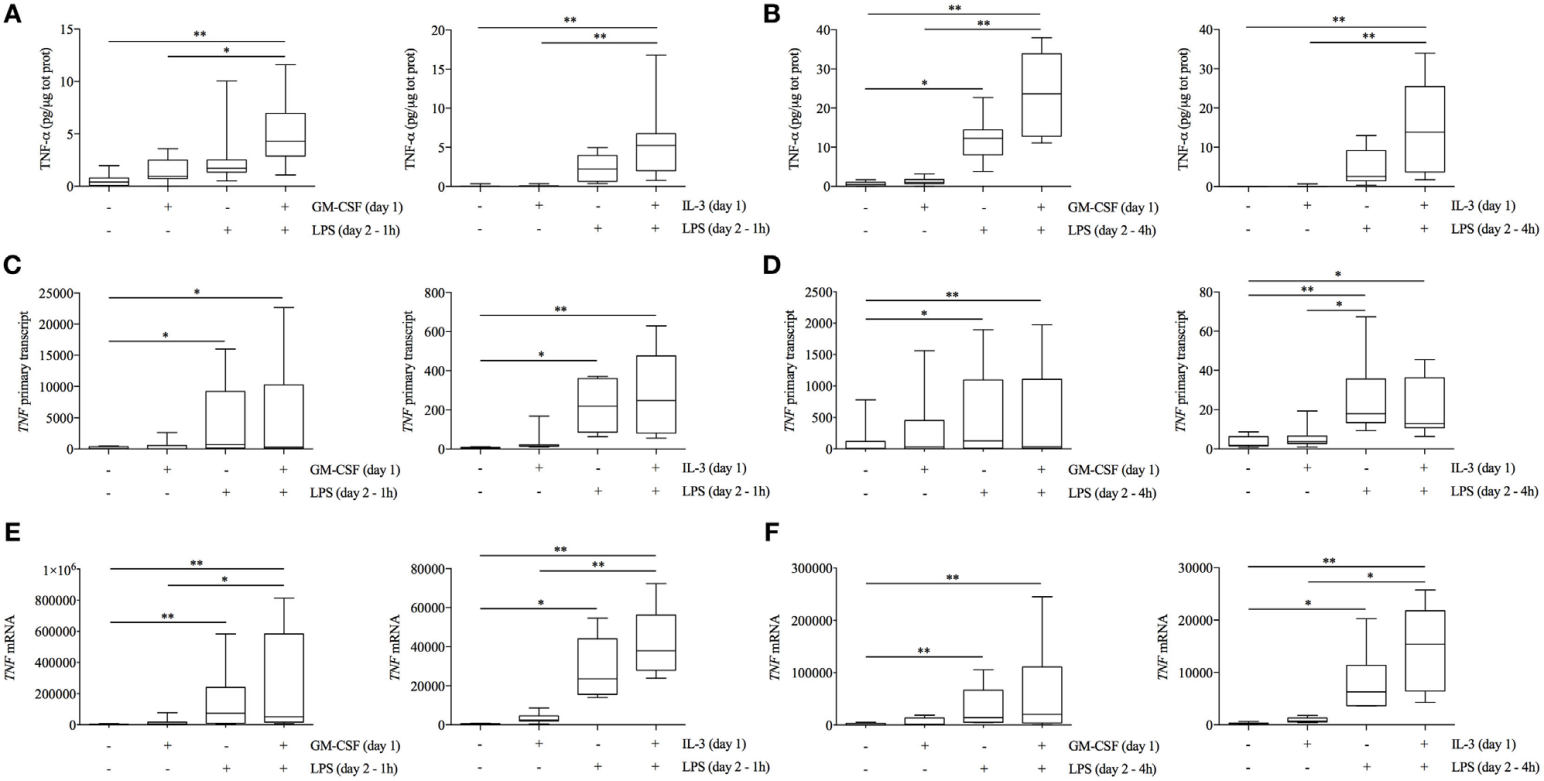
**GM-CSF and IL-3 priming modulates TNF-α protein abundance at the posttranscriptional level**. **(A–F)** Human CD14^+^ monocytes were primed with GM-CSF and IL-3 for 18 h (day 1), then washed, and stimulated with LPS (day 2) for 1 h **(A,C,E)** or 4 h **(B,D,F)**. TNF-α protein abundance **(A,B)** was measured in protein lysates by ELISA. *TNF* primary transcript **(C,D)** and mRNA **(E,F)** were measured by real-time RT-PCR. Data are shown as the median, the 25th and 75th percentiles (boxes) and 5th and 95th percentiles (whiskers) of eight independent experiments. *P < 0.05, **P < 0.01 determined by Friedman test with Dunn’s *post hoc* test.

To obtain some clue on the modulation of TNF-α in GM-CSF and IL-3 primed monocytes, we performed a time-course analysis of TNF-α intracellular protein, primary transcript (which is a more direct measure of transcription and is detected by using intronic PCR primers) (24), and mRNA levels. GM-CSF and IL-3 priming resulted in the highest TNF-α intracellular protein levels as early as after 1 h of LPS stimulation (Figure 2A). After 4 h of LPS stimulation, TNF-α intracellular protein levels were even more increased in primed monocytes (Figure 2B). The same pattern of TNF-α production was observed in cell-free supernatants (data not shown). Unexpectedly, *TNF* primary transcript (Figure 2C) and mRNA (Figure 2E) levels were either not modified or only slightly increased by GM-CSF and IL-3 priming after 1 h of LPS stimulation. After 4 h, IL-3 priming increased *TNF* mRNA levels (Figure 2F) whereas *TNF* primary transcript levels were unchanged compared to unprimed cells (Figure 2D), suggesting that IL-3 priming increases *TNF* mRNA stability. Thus, GM-CSF and IL-3 priming uncouples TNF-α protein and primary transcript levels upon LPS stimulation, thereby suggesting that TNF-α expression is modulated at the posttranscriptional level in primed monocytes.

### NAD-Dependent SIRT2 and MAPKs ERK1/2 and p38 Mediate the Effect of GM-CSF and IL-3 Priming on TNF-α Production

The expression of several genes with immune function (including TNF-α) can be regulated at the translational level, thereby modulating protein abundance independently of *de novo* transcription (33–35). A central (albeit not exclusive) role in the modulation of mRNA translation is played by the translation-initiation factor eIF4E, whose activity is regulated by mTORC1 (a multiprotein complex that includes the serine/threonine kinase mTOR and raptor) and the MAPKs ERK1/2 and p38 (34). TNF-α protein levels are also regulated by metabolic cues. Depletion of the intracellular pool of nicotinamide adenine dinucleotide (NAD) by the nicotinamide phosphoribosyltransferase (Nampt) inhibitor APO866 inhibits TNF-α protein production in response to LPS without modifying *TNF* mRNA levels. The effect of APO866 is reverted by the addition of nicotinic acid [(NA), a substrate for Nampt-independent NAD synthesis] and is mimicked by inhibitors of the NAD-dependent histone deacetylases sirtuin (SIRT) 1 and 2 (36, 37). Thus, we reasoned that these pathways could contribute to the modulation of TNF-α protein levels in GM-CSF and IL-3-primed cells and proceeded to investigate this hypothesis.

**Figure 3:**
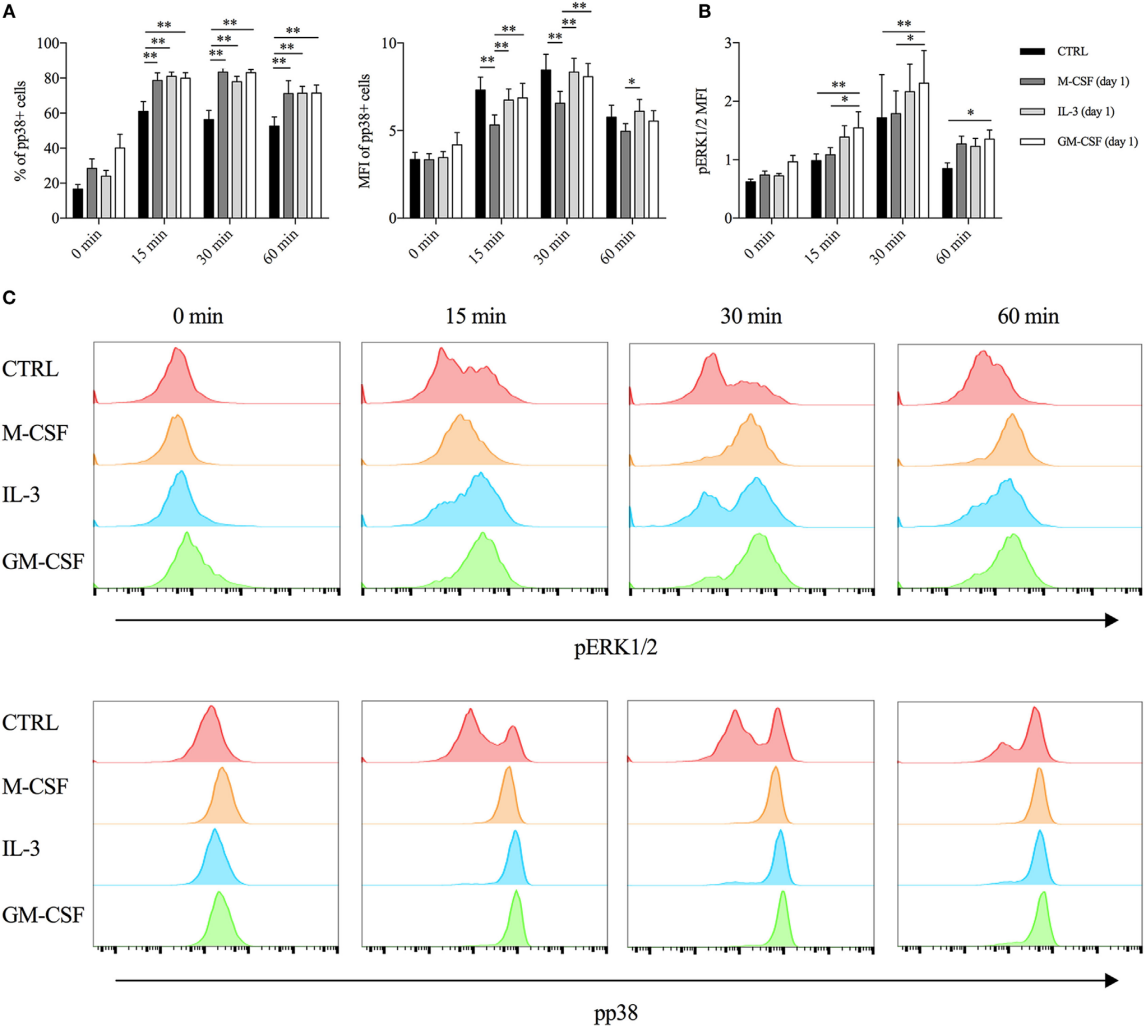
**GM-CSF and IL-3 priming increases p38 and ERK1/2 phosphorylation upon LPS stimulation**. **(A,B)** Human CD14^+^ monocytes were left unprimed (CTRL) or primed with M-CSF, IL-3, and GM-CSF for 18 h (day 1), then washed, and left untreated (0 min) or stimulated with LPS for different time points (15, 30, and 60 min). **(A)** p38 phosphorylation was assessed by flow cytometry as percentage (left panel) and median fluorescence intensity (MFI) (right panel) of pp38^+^ cells. **(B)** ERK1/2 phosphorylation was assessed by flow cytometry as MFI. **(C)** Representative histograms of p38 and ERK1/2 phosphorylation. Data are shown as mean + SEM of eight independent experiments. *P < 0.05, **P < 0.01 determined by repeated measure two-way ANOVA with Tukey’s *post hoc* test.

First, we assessed the contribution of mTORC1 by employing two different mTOR inhibitors (rapamycin and torin 1) and also an inhibitor of SIRT2 (which is involved in the regulation of the mTOR-activated translational machinery) (38). We added the inhibitors along with LPS and evaluated TNF-α intracellular protein levels after 1 h of stimulations. None of these inhibitors modulated TNF-α production in both unprimed and primed monocytes (Figure S6 in Supplementary Material). Then, we employed the same approach using inhibitors of MAPKs (i.e., ERK1/2, p38, and JNK). The p38 inhibitor SB203580 reduced TNF-α intracellular protein in both unprimed and GM-CSF or IL-3 primed cells (Figure S7A in Supplementary Material), while ERK1/2 inhibition with the MEK1/2 inhibitor U0126 was effective only in GM-CSF primed cells (Figure S7B in Supplementary Material). No effect for the JNK inhibitor SP600125 could be observed (data not shown). Importantly, ERK1/2 (Figures S7D,F in Supplementary Material) and p38 inhibition (Figures S7C,E in Supplementary Material) did not significantly modulate *TNF* RNA levels. To corroborate these findings, we performed a time-course analysis of p38 and ERK1/2 phosphorylation (pp38 and pERK1/2, respectively) upon LPS stimulation. As early as after 15 min of stimulation, GM-CSF and IL-3-primed cells exhibited the highest phosphorylation of p38, as assessed by the percentage (compared to unprimed cells) and the MFI (compared to M-CSF primed cells) of pp38^+^ cells (Figure 3A). On the contrary, only GM-CSF-primed cells exhibited increased ERK1/2 phosphorylation compared to unprimed or M-CSF-primed cells (Figures 3B,C).

**Figure 4:**
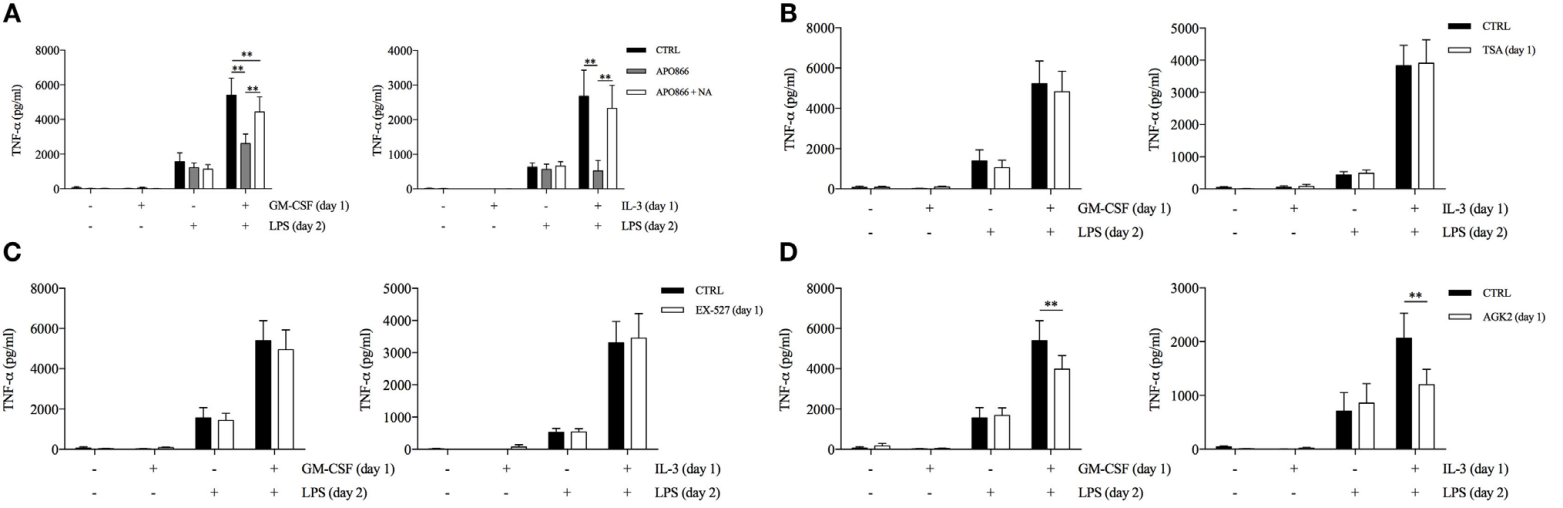
**GM-CSF and IL-3 priming requires the nicotinamide adenine dinucleotide-dependent histone deacetylase SIRT2**. **(A–D)** Human CD14^+^ monocytes were primed with GM-CSF and IL-3 for 18 h in the presence of several compounds (day 1), then washed, and stimulated with LPS for 16–18 h (day 2). TNF-α levels were assessed by ELISA in cell-free supernatants. Data are shown as mean + SEM of seven independent experiments. **P < 0.01 determined by repeated measure two-way ANOVA with Tukey’s **(A)** or Sidak’s **(B–D)** *post hoc* test.

Then, we used APO866 and NA to evaluate whether intracellular NAD levels contributed to the priming effect of GM-CSF and IL-3. APO866 concentration-dependently inhibited TNF-α secretion only when administered during priming (Figure S8A in Supplementary Material). Importantly, the addition of NA reverted the effect of APO866 (Figure 4A). We verified that APO866 reduced while NA addition restored intracellular NAD levels (Figure S8B in Supplementary Material). Interestingly, GM-CSF and IL-3 priming increased intracellular NAD levels compared to unprimed cells. However, the same effect was observed with M-CSF priming (Figure S8C in Supplementary Material), which does not modulate TNF-α secretion. We also confirmed that unprimed and M-CSF, IL-3, and GM-CSF-primed monocytes expressed comparable levels of *SIRT2* mRNA (Figure S8D in Supplementary Material). Thus, the increase in intracellular NAD levels is a condition required but not sufficient for the priming effect of GM-CSF and IL-3. We then evaluated the involvement of the NAD-dependent histone deacetylases SIRT1 and SIRT2 in GM-CSF and IL-3 priming by using the specific inhibitors EX-527 and AGK2, respectively. SIRT2 inhibition during priming selectively reduced TNF-α secretion (Figures 4C,D) and intracellular protein levels (Figure S9A in Supplementary Material) without significantly modulating *TNF* RNA (Figures S9B,C in Supplementary Material), although the effect was more pronounced for IL-3 rather than GM-CSF- primed cells. We confirmed the specificity of SIRT2 involvement by showing that the inhibitor of NAD-independent histone deacetylases TSA had no effect on GM-CSF and IL-3 priming (Figure 4B).

### GM-CSF and IL-3 Priming Modulates Monocyte Renewal in a c-Myc-Dependent Manner

**Figure 5:**
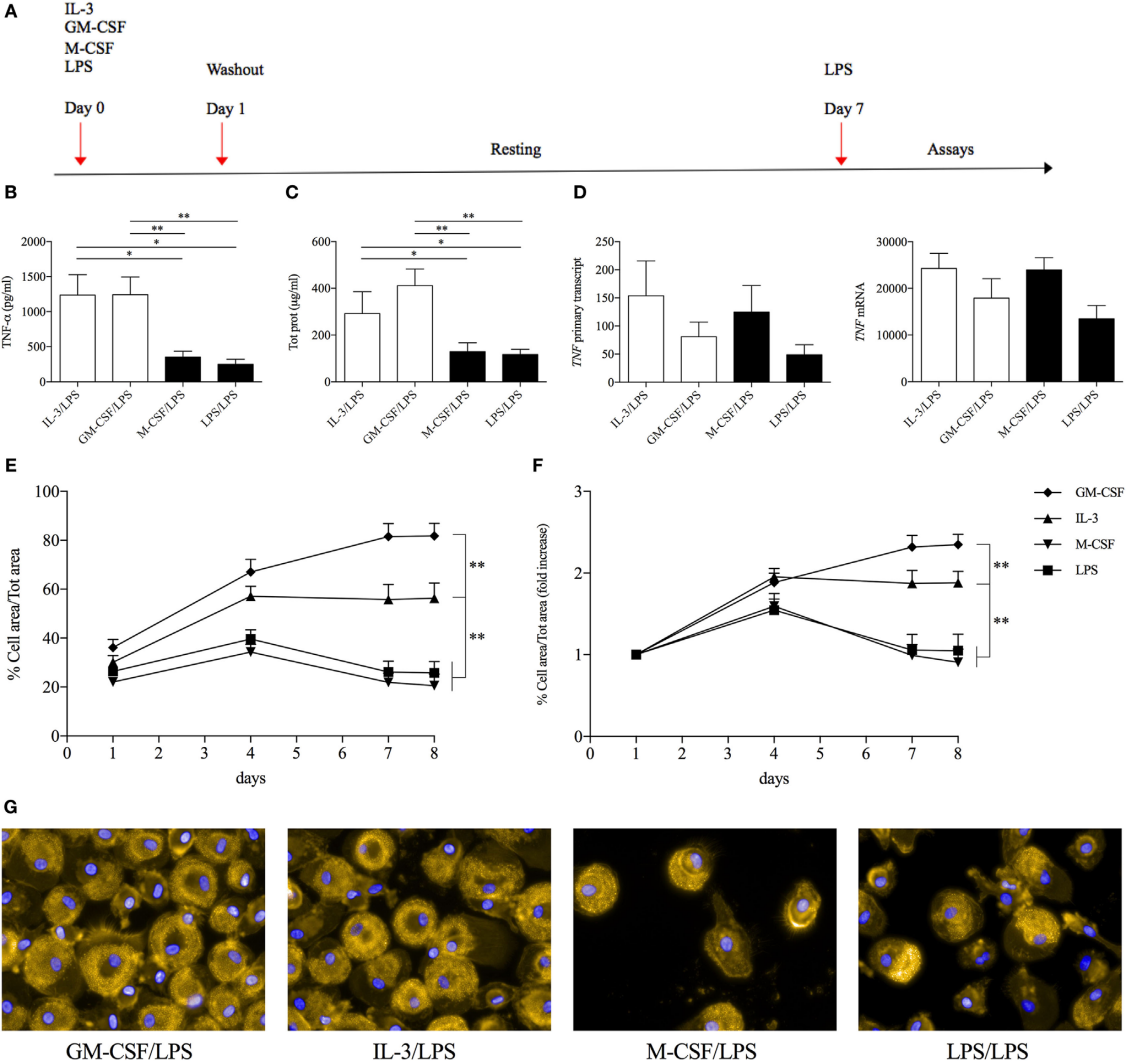
**GM-CSF and IL-3 priming induces monocyte renewal in a long-term model of trained immunity**. **(A)** Schematic representation of the long-term model of trained immunity. **(B–D)** Human CD14^+^ monocytes were treated as outlined above **(A)**. On day 7, cells were stimulated with LPS for 16–18 h **(B,C)** or 1 h **(D)**. TNF-α levels were assessed by ELISA in cell-free supernatants **(B)**. Cells were harvested for total protein quantification **(C)** or RNA extraction and evaluation of *TNF* primary transcript and mRNA levels by real time RT-PCR **(D)**. **(E–G)** Human CD14^+^ monocytes were treated as outlined above **(A)** and on day 7 were stimulated with LPS for 16–18 h. Brightfield images (15 fields/well) were obtained at the indicated time points with a 20× objective. The percentage of cell area/total area was calculated and expressed as absolute values **(E)** or fold increase over day 1 **(F)**. On day 8, representative fluorescence microscopy images were also obtained with a 60× objective. Orange, actin; blue, nuclei **(G)**. Data are shown as mean + SEM of 10 **(B,C)**, five **(D)**, or eight **(E,F)** independent experiments. *P < 0.05, **P < 0.01 determined by repeated measure one-way **(B–D)** or two-way **(E,F)** ANOVA with Tukey’s *post hoc* test.

The results obtained so far have characterized the role of GM-CSF and IL-3 in a short-term model of trained immunity. Since the effects of training can be retained by human monocytes for several days even in the absence of the training stimulus (e.g., β-glucan) (26, 29), we investigated the possible roles of GM-CSF and IL-3 in a long-term model of trained immunity. Monocytes were primed with GM-CSF, IL-3, M-CSF, and LPS for 16–18 h, washed out, and rested in complete medium until day 7 when they were stimulated with LPS (Figure 5A). In accordance with the results obtained with β-glucan, cells primed with GM-CSF or IL-3 secreted higher levels of TNF-α compared to those primed with M-CSF and LPS (Figure 5B). Although these results could suggest stable chromatin remodeling events in response to GM-CSF and IL-3 priming (similar to what has been demonstrated for β-glucan), we also found that GM-CSF and IL-3 priming increased total cellular protein content (Figure 5C) and that monocytes primed with GM-CSF, IL-3, and M-CSF expressed comparable levels of *TNF* RNA upon LPS stimulation (Figure 5D). Thus, we reasoned that GM-CSF and IL-3 priming could induce monocyte renewal and increase the number of cells, which in turn would result in higher TNF-α secretion upon LPS stimulation. We investigated this hypothesis by evaluating the degree of cell confluency (assessed as the proportion of cell area to total area). Monocytes primed with GM-CSF and IL-3 reached remarkable higher cell confluency on day 7 compared to monocyte primed with M-CSF or LPS (Figure 5E). Comparable results were obtained when we expressed cell confluency on days 4, 7, and 8 as fold increase over day 1 to account for possible differences in cell detachment after the washing step (Figure 5F). We also obtained evidence of increased cell number following GM-CSF and IL-3 priming by fluorescence (Figure 5G) and time-lapse microscopy (Videos S1–S4 in Supplementary Material). Thus, GM-CSF and IL-3 priming induces monocyte renewal in a long-term model of trained immunity.

**Figure 6:**
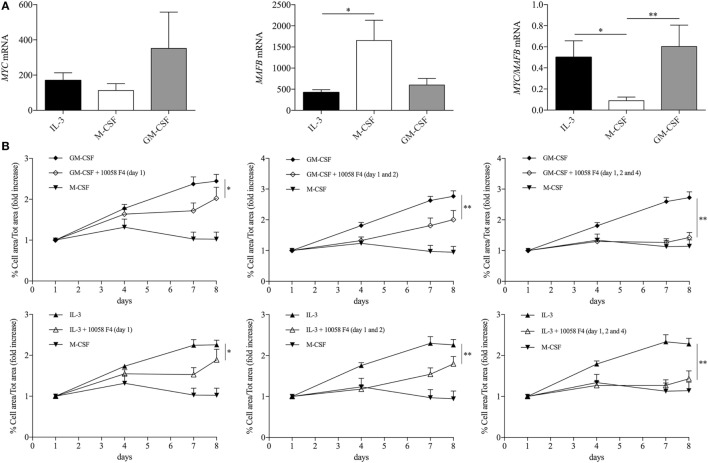
**GM-CSF and IL-3 elicited monocyte renewal requires c-Myc**. **(A)** Human CD14^+^ monocytes were primed with IL-3, M-CSF, or GM-CSF for 18 h. Then, cells were harvested for RNA isolation and *MYC* and *MAFB* mRNA levels were evaluated by real-time RT-PCR. **(B)** Human CD14^+^ monocytes were treated as outlined in Figure 5A using GM-CSF, IL-3, and M-CSF as priming stimuli. Cells were also treated with the c-Myc inhibitor 10058 F4 on day 1 (left panels), day 1 and 2 (middle panels), day 1, 2, and 4 (right panels). Analysis was performed as in Figure 5F. Data are shown as mean + SEM of seven **(A)** or eight **(B)** independent experiments. *P < 0.05, **P < 0.01 determined by repeated measure one-way **(A)** or two-way **(B)** ANOVA with Tukey’s *post hoc* test.

**Figure 7:**
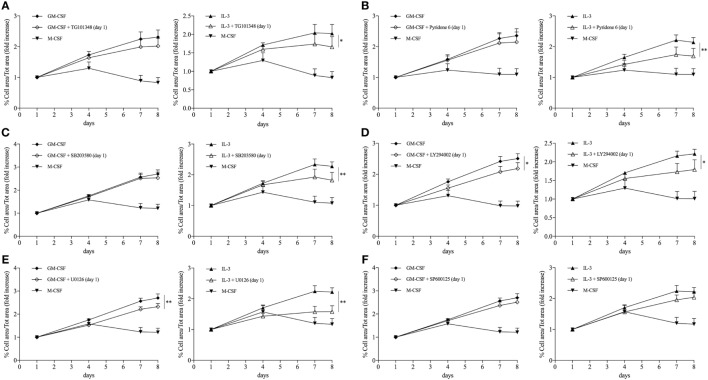
**GM-CSF and IL-3 priming induces monocyte renewal through different signaling pathways**. **(A–F)** Human CD14^+^ monocytes were treated as outlined in Figure 5A using GM-CSF and IL-3 as priming stimuli. Cells were also treated with different inhibitors during the priming phase. Analysis was performed as in Figure 5F. Data are shown as mean + SEM of eight independent experiments. *P < 0.05, **P < 0.01 determined by repeated measure two-way ANOVA with Tukey’s *post hoc* test.

Maintenance of the pool of tissue-resident macrophages is supported by self-renewal without the contribution of hematopoietic stem cell in several experimental models (39–51). Interestingly, macrophage renewal is sustained by a gene network that includes the transcription factor c-Myc and requires downregulation of the transcription factor MafB (49, 52, 53). In keeping with these data, GM-CSF and IL-3 priming increased the *MYC*/*MAFB* mRNA ratio compared to M-CSF priming (Figure 6A). Moreover, the c-Myc inhibitor 10058 F4 restrained monocyte renewal induced by GM-CSF and IL-3 priming (Figure 6B). To better characterize the pathways required for GM-CSF- and IL-3-induced monocyte renewal, several inhibitors were added along with GM-CSF or IL-3 and then cell confluency was evaluated as indicated above. Metabolic inhibitors (2-Deoxy-d-Glucose, etomoxir, APO866) (Figure S10 in Supplementary Material) as well as the JNK inhibitor SP600125 (Figure 7F) had no effect on cell confluency. The selective JAK2 inhibitor TG101348 (Figure 7A), the pan-JAK inhibitor Pyridone 6 (Figure 7B), and the p38 inhibitor SB203580 (Figure 7C) restrained only IL-3 priming-induced monocyte expansion. Finally, the PI3K inhibitor LY294002 (Figure 7D) and the MEK1/2 inhibitor U0126 (which in turn inhibits ERK1/2 activity) (Figure 7E) reduced monocyte expansion in response to both GM-CSF and IL-3 priming, although IL-3 priming was affected to a greater degree.

## Discussion

Here, we show that GM-CSF and IL-3, hematopoietic cytokines that play a central role in regulating myeloid cell expansion and effector response in several *in vivo* models of inflammation and autoimmunity, modulate human CD14^+^ monocyte response in short- and long-term models of trained immunity. To understand the regulation of TNF-α production in primed monocytes, we compared intracellular TNF-α protein levels, *TNF* mRNA, and primary transcript levels upon LPS stimulation for 1 and 4 h. Although in several experimental models priming of monocyte and macrophages results in chromatin remodeling (20, 22–24, 26–30), our data support a posttranscriptional regulation of TNF-α production in primed monocytes. Posttranscriptional mechanisms, including modulation of mRNA stability and translation (33–35), are increasingly recognized as critical regulators of gene and protein expression also in immune cells (25, 34, 36, 37, 54, 55). Our results demonstrate that inhibition of SIRT2 during priming and of p38 or ERK1/2 (the latter only for GM-CSF primed cells) during LPS stimulation reduces intracellular TNF-α protein levels without significantly modulating *TNF* RNA levels. In keeping with these data, GM-CSF priming increases phosphorylation of both ERK1/2 and p38 upon LPS stimulation, while IL-3 priming enhances only p38 phosphorylation. However, it is still unclear how SIRT2 inhibition during the priming phase modulates TNF-α protein production. We verified that SIRT2 inhibition during the priming phase does not reduce ERK1/2 and p38 phosphorylation upon LPS stimulation (Figures S11A,B in Supplementary Material). Nevertheless, the phenotype of GM-CSF- and IL-3-primed monocytes may be regulated by pathways not investigated in this study. Apart from the molecular mechanisms that may also reveal subtle differences in monocyte responsiveness to GM-CSF and IL-3 priming, genome-wide approaches aimed at evaluating the translatome or at comparing the transcriptome and the proteome may provide critical insights into the modulation of LPS-induced monocyte response by GM-CSF and IL-3 priming.

In order to fully characterize the priming effect of GM-CSF and IL-3, we also employed a long-term model of trained immunity. Using this model, it was shown that human monocyte primed with β-glucan undergo extensive chromatin remodeling that modulate their response to subsequent microbial challenges (e.g., increased TNF-α production upon LPS stimulation) (26, 29). Our results demonstrate that GM-CSF and IL-3 priming increases LPS-induced TNF-α production in this model, but the mechanism is independent of increased *TNF* gene expression (and likely chromatin remodeling). Instead, GM-CSF and IL-3 priming induces monocyte renewal by increasing the *MYC*/*MAFB* mRNA ratio. Interestingly, proliferation of monocyte-derived cells (either macrophages or dendritic cells) has been observed in several models of inflammation (49, 56, 57). Although we found similarities as well as differences between GM-CSF and IL-3 priming in the pathways required for monocyte renewal, it is noteworthy that GM-CSF priming induces a greater monocyte expansion than IL-3 priming. The reason for this difference probably relies in the constitutive expression of CD116 (the α subunit of GM-CSF receptor) by human monocytes (58), while CD123 (the α subunit of IL-3 receptor) expression is inducible (16, 59). Nevertheless, bone marrow CD14^+^ monocytes express CD123 (60), thus it is tempting to speculate that IL-3 preferentially acts on bone marrow-resident monocytes, while GM-CSF exerts its effect on peripheral blood monocytes. Moreover, since priming with both cytokines induces monocyte renewal and priming also affects the biology of hematopoietic precursors (61–63), it would be interesting to assess whether GM-CSF and IL-3 priming can modulate the functions of hematopoietic precursors and their progeny. Further studies are required to address these questions.

In conclusion, we characterize the activation of human monocytes in response to the hematopoietic cytokines GM-CSF and IL-3 using *in vitro* models of trained immunity. Our results point to mechanisms of GM-CSF and IL-3 priming that are independent of chromatin remodeling, but unexpectedly rely on posttranscriptional regulation of TNF-α production and c-Myc-dependent monocyte renewal. Given the central role of GM-CSF and IL-3 in inflammatory and autoimmune diseases (6–11), our results provide important information to understand mechanisms of monocyte activation in such disorders and prompt their investigation *in vivo*. We also demonstrate that GM-CSF and IL-3 activate monocytes through shared as well as distinct pathways. Such differences may become even more evident *in vivo*, since it is conceivable that GM-CSF and IL-3 are produced with a different timing and/or act on different target populations (e.g., bone marrow vs. peripheral cells). Thus, GM-CSF and IL-3 may differentially impact on the immune response due to distinct effects at the cellular and the system levels. Understanding such complexity is poised to unravel distinct as well as overlapping roles for GM-CSF and IL-3 in health and disease.

## Author Contributions

Participated in research design: FB, RI, SS, SL, ES, MG, GV, FG, GP, and GM; conducted experiments: FB, RI, SS, SL, ES, and MG; performed data analysis: FB, RI, SS; wrote or contributed to the writing the manuscript: FB, RI, SS, and GM.

## Conflict of Interest Statement

The authors declare that the research was conducted in the absence of any commercial or financial relationships that could be construed as a potential conflict of interest.

## Supplementary Material

Videos S1–S4 **GM-CSF and IL-3 priming induces monocyte renewal in a long-term model of trained immunity**. Human CD14^+^ monocytes were treated as outlined in Figure 5A using GM-CSF (Video S1 in Supplementary Material), IL-3 (Video S2 in Supplementary Material), M-CSF (Video S3 in Supplementary Material), and LPS (Video S4 in Supplementary Material) as priming stimuli. From day 1 to 7, digital phase contrast images of 15 fields/well were taken every 60 min with a 20× objective. Representative videos of single fields are shown for each experimental condition.
