# Supplementary material for: GM-CSF and IL-3 Modulate Human Monocyte TNF-α Production and Renewal in In Vitro Models of Trained Immunity
Source: Front Immunol. 2017 Jan 16;7:680. doi: 10.3389/fimmu.2016.00680 (PMC5237654; doi:10.3389/fimmu.2016.00680)
Supplement: Supplementary file 1 [file data_sheet_1.pdf]

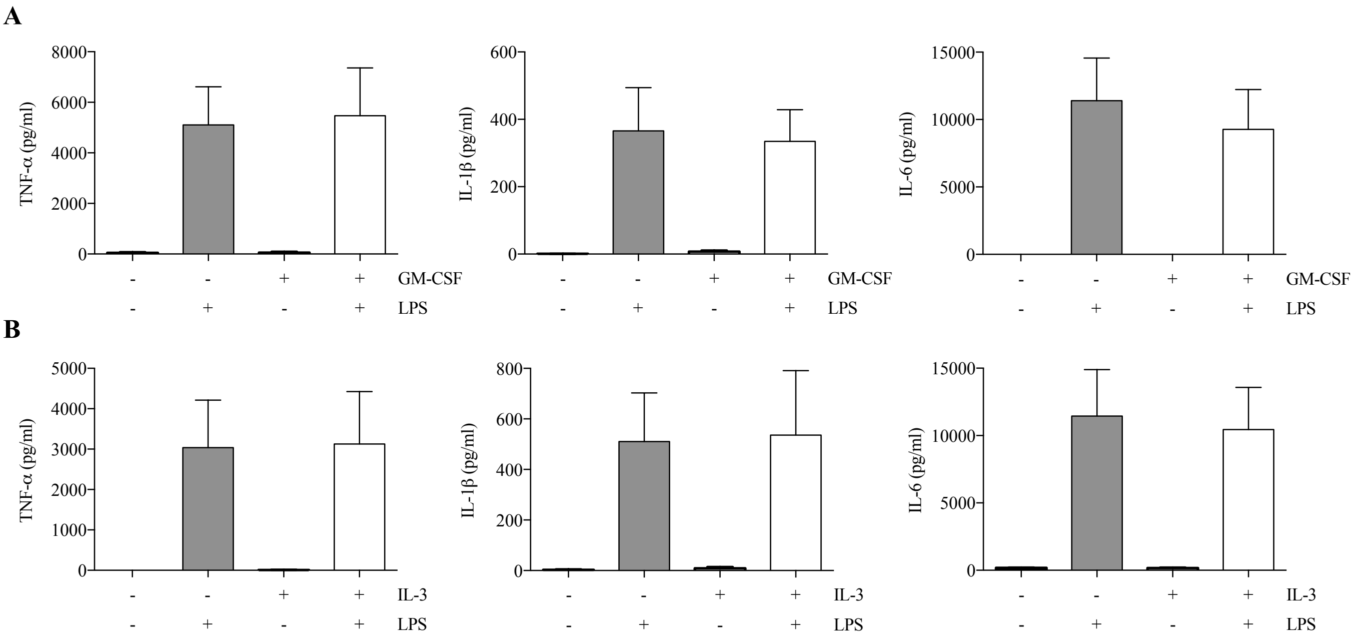
A
TNF-α (pg/ml)
8000
6000
4000
2000
0
IL-1β (pg/ml)
600
400
200
0
IL-6 (pg/ml)
15000
10000
5000
0
- - + + GM-CSF
- + - + LPS
B
TNF-α (pg/ml)
5000
4000
3000
2000
1000
0
IL-1β (pg/ml)
800
600
400
200
0
IL-6 (pg/ml)
15000
10000
5000
0
- - + + IL-3
- + - + LPS

Figure S1

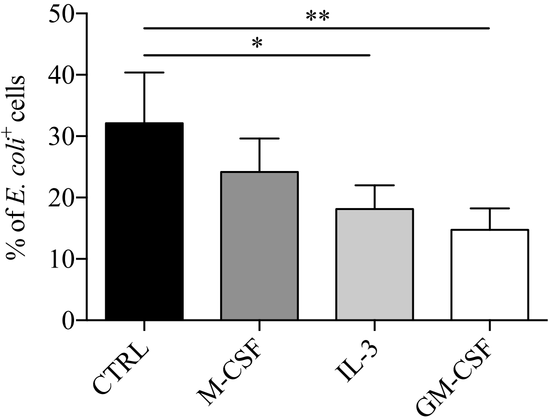

Figure S2

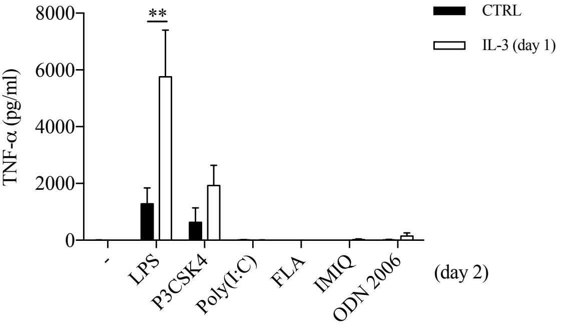
8000
6000
4000
2000
0
TNF-α (pg/ml)
**
CTRL
IL-3 (day 1)
-
LPS
P3CSK4
Poly(I:C)
FLA
IMIQ
ODN 2006
(day 2)

Figure S3

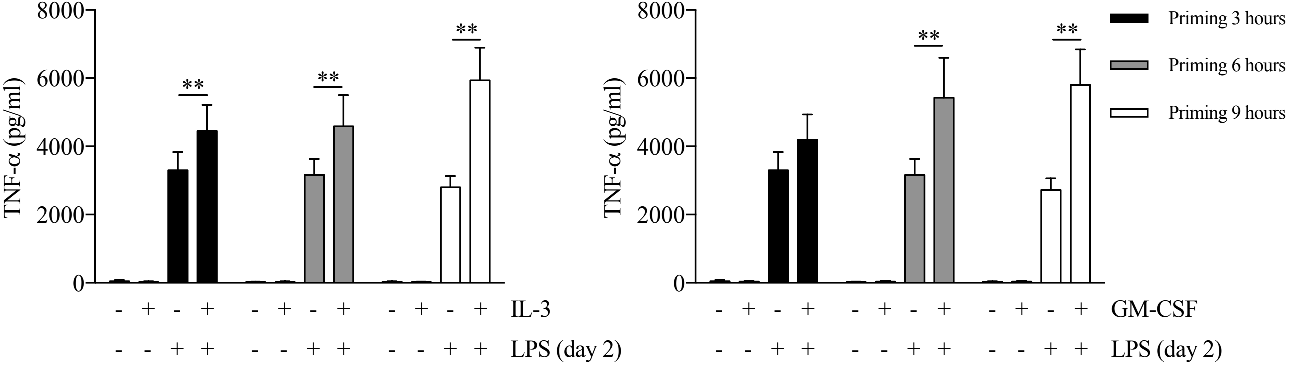
8000
6000
4000
2000
0
TNF-α (pg/ml)
**
IL-3
LPS (day 2)
GM-CSF
Priming 3 hours
Priming 6 hours
Priming 9 hours

Figure S4

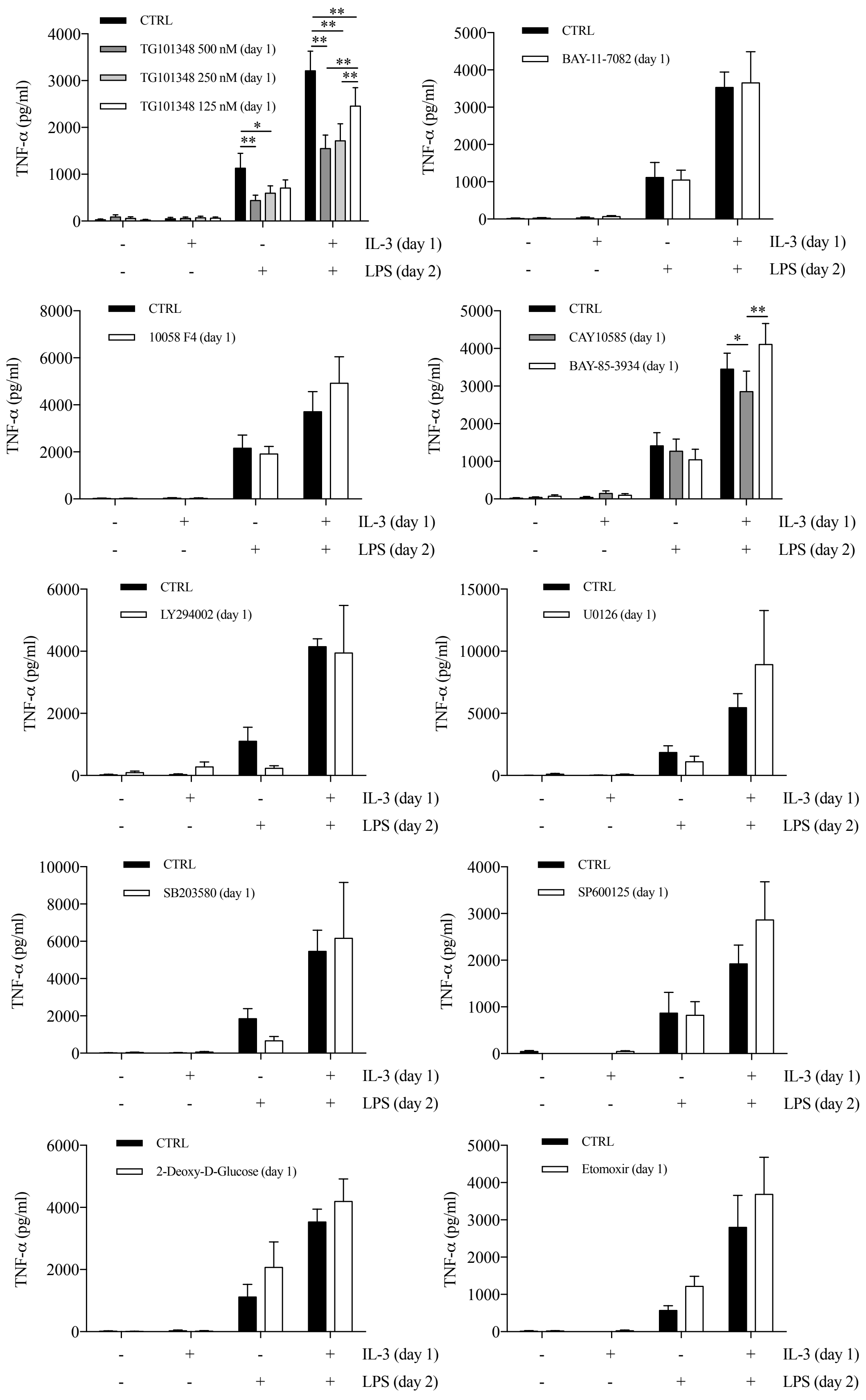
TNF-α (pg/ml)
CTRL
TG101348 500 nM (day 1)
TG101348 250 nM (day 1)
TG101348 125 nM (day 1)
BAY-11-7082 (day 1)
10058 F4 (day 1)
CAY10585 (day 1)
BAY-85-3934 (day 1)
LY294002 (day 1)
U0126 (day 1)
SB203580 (day 1)
SP600125 (day 1)
2-Deoxy-D-Glucose (day 1)
Etomoxir (day 1)
IL-3 (day 1)
LPS (day 2)

Figure S5

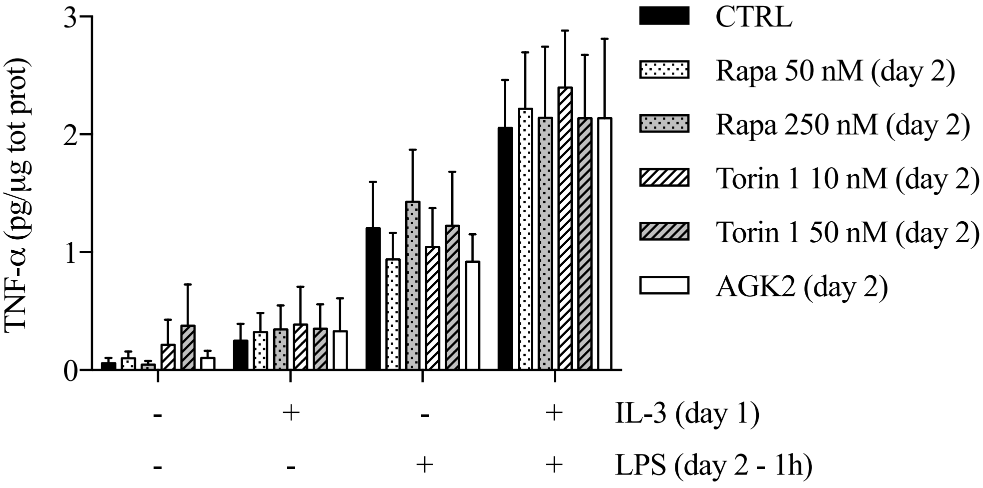
TNF-α (pg/µg tot prot)
3
2
1
0
CTRL
Rapa 50 nM (day 2)
Rapa 250 nM (day 2)
Torin 1 10 nM (day 2)
Torin 1 50 nM (day 2)
AGK2 (day 2)
- + - +
IL-3 (day 1)
- - + +
LPS (day 2 - 1h)

Figure S6

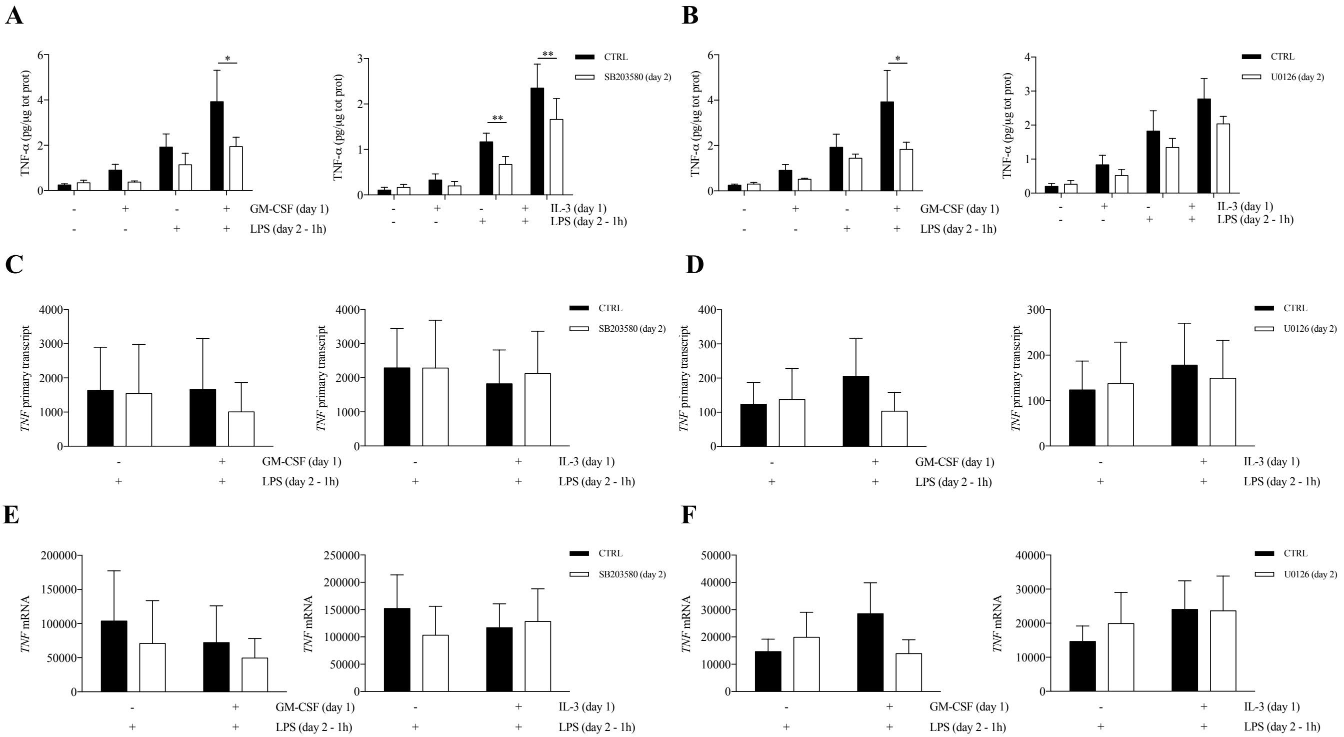
A
B
C
D
E
F
TNF-α (pg/µg tot prot)
TNF primary transcript
TNF mRNA
CTRL
SB203580 (day 2)
U0126 (day 2)
GM-CSF (day 1)
IL-3 (day 1)
LPS (day 2 - 1h)
*
**

Figure S7

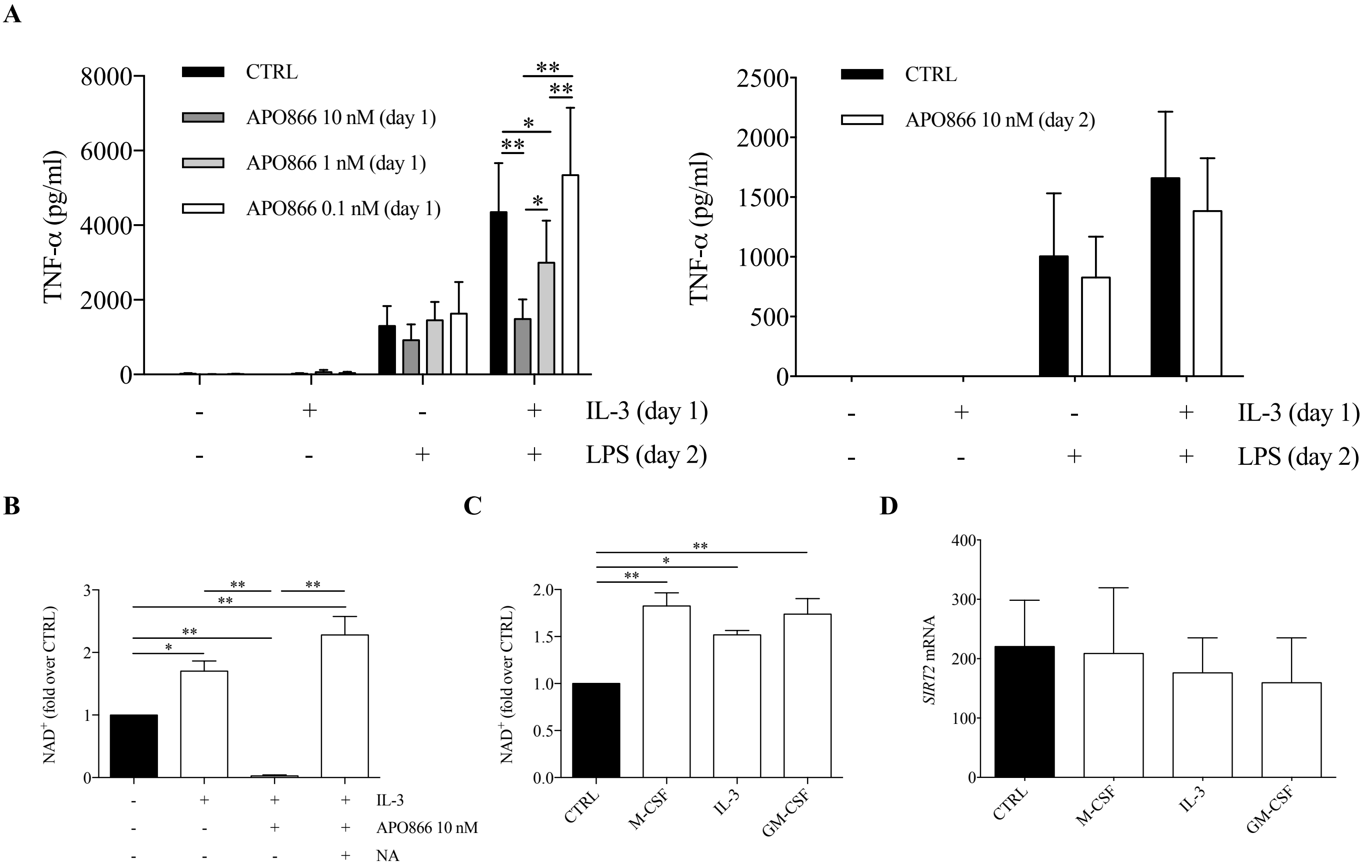
A
CTRL
APO866 10 nM (day 1)
APO866 1 nM (day 1)
APO866 0.1 nM (day 1)
TNF-α (pg/ml)
8000
6000
4000
2000
0
IL-3 (day 1)
LPS (day 2)
APO866 10 nM (day 2)
2500
2000
1500
1000
500
B
NAD+ (fold over CTRL)
IL-3
APO866 10 nM
NA
C
CTRL
M-CSF
IL-3
GM-CSF
D
SIRT2 mRNA
400
300
200
100

Figure S8

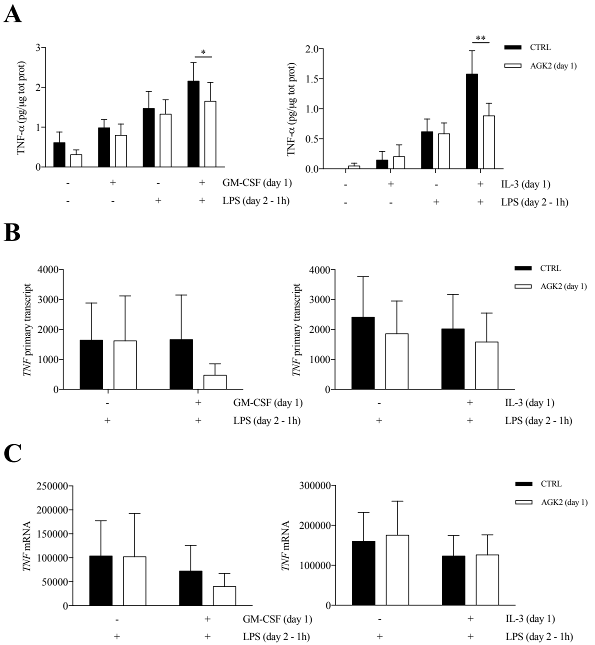
A
TNF-α (pg/µg tot prot)
GM-CSF (day 1)
LPS (day 2 - 1h)
CTRL
AGK2 (day 1)
IL-3 (day 1)
B
TNF primary transcript
C
TNF mRNA

Figure S9

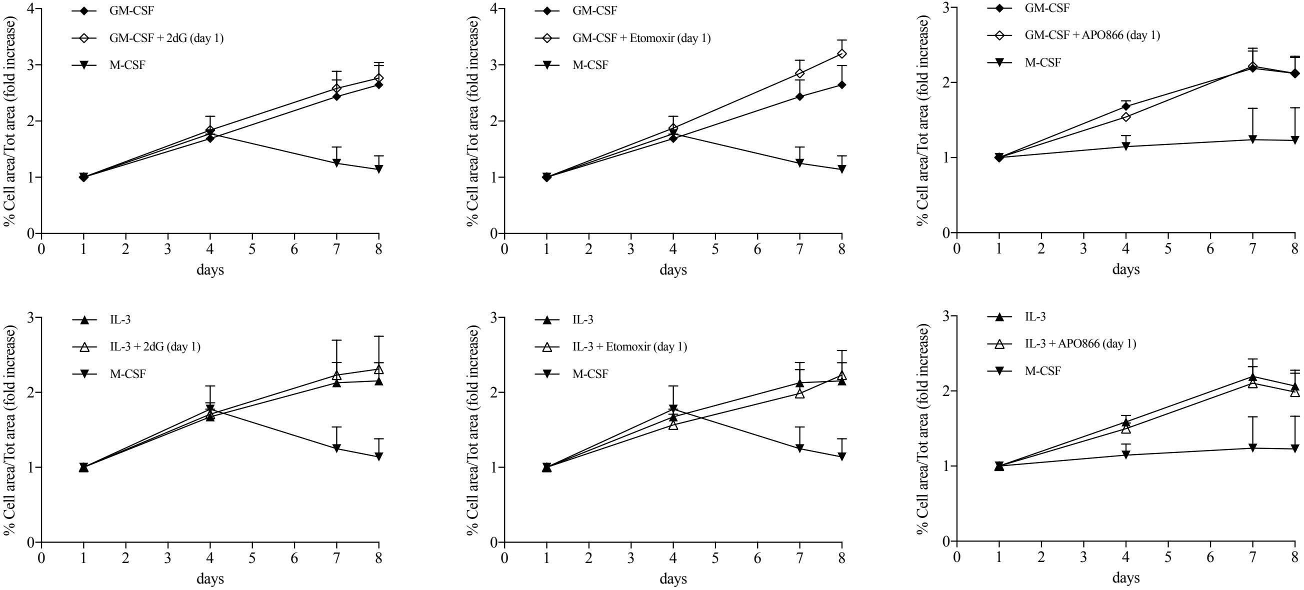
GM-CSF
GM-CSF + 2dG (day 1)
M-CSF
% Cell area/Tot area (fold increase)
days
GM-CSF
GM-CSF + Etomoxir (day 1)
M-CSF
% Cell area/Tot area (fold increase)
days
GM-CSF
GM-CSF + APO866 (day 1)
M-CSF
% Cell area/Tot area (fold increase)
days
IL-3
IL-3 + 2dG (day 1)
M-CSF
% Cell area/Tot area (fold increase)
days
IL-3
IL-3 + Etomoxir (day 1)
M-CSF
% Cell area/Tot area (fold increase)
days
IL-3
IL-3 + APO866 (day 1)
M-CSF
% Cell area/Tot area (fold increase)
days

Figure S10

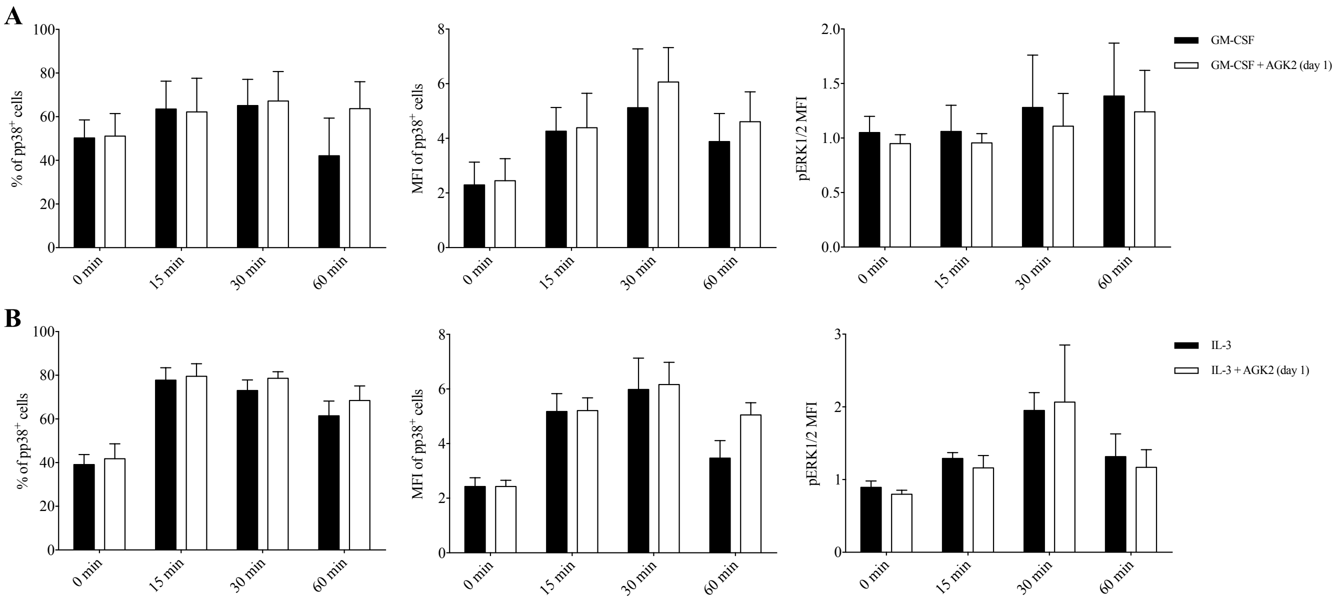

A
100
80
60
40
20
0
% of pp38+ cells
0 min
15 min
30 min
60 min
8
6
4
2
0
MFI of pp38+ cells
2.0
1.5
1.0
0.5
0.0
pERK1/2 MFI
GM-CSF
GM-CSF + AGK2 (day 1)
B
3
2
1
0
IL-3
IL-3 + AGK2 (day 1)

Figure S11

Figure S1. Simultaneous addition of GM-CSF or IL-3 to LPS does not modulate cytokine production. (A, B) Human CD14$^+$ monocytes were stimulated with LPS and GM-CSF (A) or IL-3 (B) for 16-18 hours. TNF-α, IL-1β and IL-6 levels were assessed by ELISA in cell-free supernatants. Data are shown as mean + SEM of 5 (A) or 6 (B) independent experiments and were analyzed by repeated measure one-way ANOVA with Tukey's post hoc test.

Figure S2. GM-CSF and IL-3 priming does not enhance *E. coli* phagocytosis. Human CD14$^+$ monocytes were left untreated or primed with M-CSF, IL-3 and GM-CSF for 18 hours. Then, pHrodo *E. coli* BioParticles were added for 1 hour and the percentage of phagocytic cells was measured with the Operetta High-Content Imaging System (PerkinElmer) as indicated in the Materials and methods section. Data are shown as mean + SEM of 7 independent experiments. * $P < .05$, ** $P < .01$ determined by repeated measure one-way ANOVA with Tukey's post hoc test.

Figure S3. LPS stimulation induces the highest TNF-α production by primed monocytes. Human CD14$^+$ monocytes were left unprimed (CTRL) or primed with IL-3 for 18 hours (day 1), then washed and stimulated with several TLR agonists for 16-18 hours (day 2). TNF-α levels were assessed by ELISA in cell-free supernatants. Data are shown as mean + SEM of 4 independent experiments. ** $P < .01$ determined by repeated measure two-way ANOVA with Sidak's post hoc test.

Figure S4. Time-course analysis of GM-CSF and IL-3 priming. Human CD14$^+$ monocytes were primed with IL-3 (left panel) or GM-CSF (right panel) for different times as indicated in the figure, then washed and left untreated or stimulated with LPS for 16-18 hours (day 2). TNF-α levels were assessed by ELISA in cell-free supernatants. Data are shown as mean + SEM of 8 independent experiments. ** $P < .01$ determined by repeated measure two-way ANOVA with Tukey's post hoc test.

Figure S5. Molecular pathway analysis of monocyte priming. Human CD14$^+$ monocytes were primed with IL-3 and the indicated compounds for 18 hours (day 1), then washed and left untreated or stimulated with LPS for 16-18 hours (day 2). TNF-α levels were assessed by ELISA in cell-free supernatants. Data are shown as mean + SEM of 4-10 independent experiments. * $P < .05$, ** $P < .01$ determined by repeated measure two-way ANOVA with Tukey's or Sidak's post hoc test.

Figure S6. mTOR does not regulate TNF-α protein production. Human CD14$^+$ monocytes were primed with IL-3 and the indicated compounds for 18 hours (day 1), then washed and left untreated or stimulated with LPS for 1 hour (day 2). TNF-α levels were assessed by ELISA in total protein lysates. Data are shown as mean + SEM of 4 independent experiments. Data were analyzed by repeated measure two-way ANOVA with Tukey's post hoc test.

Figure S7. p38 and ERK1/2 modulate TNF-α protein production in primed monocytes. (A-F) Human CD14$^+$ monocytes were primed with GM-CSF and IL-3 for 18 hours (day 1), then washed and left untreated or stimulated with LPS for 1 hour in the presence or absence of the p38 inhibitor SB203580 (A, C, E) or the MEK1/2 inhibitor U0126 (B, D, F) (day 2). TNF-α levels were assessed by ELISA in total protein lysates (A, B). Alternatively, cells were harvested for RNA extraction and evaluation of *TNF* primary transcript (C, D) and mRNA (E, F) levels by real time RT-PCR. Data are shown as mean + SEM of 4 (A, B) or 6 (C-F) independent experiments. * $P < .05$, ** $P < .01$ determined by repeated measure two-way ANOVA with Sidak's post hoc test.

Figure S8. NAD metabolism and monocyte priming. (A) Human CD14$^+$ monocytes were primed with IL-3 in the presence (left panel) or absence (right panel) of the Nampt inhibitor APO866 for 18 hours (day 1), then washed and stimulated with LPS in the absence (left panel) or presence (right

panel) of APO866 for 16-18 hours (day 2). TNF-α levels were assessed by ELISA in cell-free supernatants. (B-D) Cells were left untreated (CTRL), stimulated with IL-3 in the presence of APO866 and nicotinic acid (NA) (B), or with M-CSF, IL-3 and GM-CSF (C, D) for 18 hours. Intracellular NAD levels were measured in cellular lysates and expressed as fold over CTRL (B, C). Alternatively, cells were harvested for RNA extraction and evaluation of *SIRT2* mRNA levels by real time RT-PCR (D). Data are shown as mean + SEM of 6 (A-C) or 4 (D) independent experiments. * $P < .05$, ** $P < .01$ determined by repeated measure two-way ANOVA with Tukey's or Sidak's post hoc test (A, respectively left and right panel) or repeated measure one-way ANOVA with Tukey's post hoc test (B-D).

Figure S9. SIRT2 modulate TNF-α protein production in primed monocytes. (A-C) Human $CD14^+$ monocytes were primed with GM-CSF and IL-3 for 18 hours in the presence or absence of the SIRT2 inhibitor AGK2 (day 1), then washed and left untreated or stimulated with LPS for 1 hour (day 2). TNF-α levels were assessed by ELISA in total protein lysates (A). Alternatively, cells were harvested for RNA extraction and evaluation of *TNF* primary transcript (B) and mRNA (C) levels by real time RT-PCR. Data are shown as mean + SEM of 4 (A) or 6 (B, C) independent experiments. * $P < .05$, ** $P < .01$ determined by repeated measure two-way ANOVA with Sidak's post hoc test.

Figure S10. Metabolic inhibitors do not modulate monocyte renewal. Human $CD14^+$ monocytes were treated as outlined in Fig. 5A using GM-CSF (upper panels) and IL-3 (lower panels) as priming stimuli. Cells were also treated with different inhibitors during the priming phase. Analysis was performed as in Fig. 5F. Data are shown as mean + SEM of 8 independent experiments. Data were analysed by repeated measure two-way ANOVA with Tukey's post hoc test.

Figure S11. SIRT2 inhibition during the priming phase does not modulate LPS-induced p38 and ERK1/2 phosphorylation. (A, B) Human $CD14^+$ monocytes were primed with GM-CSF (A) and IL-3 (B) in the presence or absence of the SIRT2 inhibitor AGK2 for 18 hours (day 1), then washed and left untreated (0 min) or stimulated with LPS for different timepoints (15 min, 30 min, 60 min). p38 phosphorylation was assessed by flow cytometry as percentage (left panel) and MFI (right panel) of $pp38^+$ cells. ERK1/2 phosphorylation was assessed by flow cytometry as MFI. Data are shown as mean + SEM of 4 independent experiments. Data were analyzed by repeated measure two-way ANOVA with Sidak's post hoc test.

Video 1-4. GM-CSF and IL-3 priming induces monocyte renewal in a long-term model of trained immunity. Human $CD14^+$ monocytes were treated as outlined in Fig. 5A using GM-CSF (Video 1), IL-3 (Video 2), M-CSF (Video 3) and LPS (Video 4) as priming stimuli. From day 1 to 7 digital phase contrast images of 15 fields/well were taken every 60 minutes with a 20x objective. Representative videos of single fields are shown for each experimental condition.
